# On the ease of predicting the thermodynamic properties of beta-cyclodextrin inclusion complexes

**DOI:** 10.1186/1752-153X-1-29

**Published:** 2007-11-15

**Authors:** Andreas Steffen, Joannis Apostolakis

**Affiliations:** 1 Max-Planck-Institut für Informatik, Computational Biology and Applied Algorithmics, Stuhlsatzenhausweg 85, 66123 Saarbrücken, Germany; 2 Ludwig-Maximilians-Universität München, Institut für Informatik, Lehr- und Forschungseinheit für Bioinformatik, Amalienstrasse 17, 80333 München, Germany

## Abstract

**Background:**

In this study we investigated the predictability of three thermodynamic quantities related to complex formation. As a model system we chose the host-guest complexes of *β*-cyclodextrin (*β*-CD) with different guest molecules. A training dataset comprised of 176 *β*-CD guest molecules with experimentally determined thermodynamic quantities was taken from the literature. We compared the performance of three different statistical regression methods – principal component regression (PCR), partial least squares regression (PLSR), and support vector machine regression combined with forward feature selection (SVMR/FSS) – with respect to their ability to generate predictive quantitative structure property relationship (QSPR) models for ΔG°, ΔH° and ΔS° on the basis of computed molecular descriptors.

**Results:**

We found that SVMR/FFS marginally outperforms PLSR and PCR in the prediction of Δ*G°*, with PLSR performing slightly better than PCR. PLSR and PCR proved to be more stable in a nested cross-validation protocol. Whereas Δ*G° *can be predicted in good agreement with experimental values, none of the methods led to comparably good predictive models for Δ*H°*. In using the methods outlined in this study, we found that Δ*S° *appears almost unpredictable. In order to understand the differences in the ease of predicting the quantities, we performed a detailed analysis. As a result we can show that free energies are less sensitive (than enthalpy or entropy) to the small structural variations of guest molecules. This property, as well as the lower sensitivity of Δ*G° *to experimental conditions, are possible explanations for its greater predictability.

**Conclusion:**

This study shows that the ease of predicting Δ*G° *cannot be explained by the predictability of either Δ*H° *or ΔS°. Our analysis suggests that the poor predictability of *TΔS° *and, to a lesser extent, Δ*H° *has to do with a stronger dependence of these quantities on the structural details of the complex and only to a lesser extent on experimental error.

## Background

Cyclodextrins (CDs) are cyclic oligomers of *α*-D-Glucose, which can be categorised into four types: *α*-, *β*-, *γ*- and *δ*-CDs, corresponding to 6, 7, 8 or 9 *α*-D-glucose units. The shape of CDs has been described as torus- or doughnut-like, reflecting the existence of a cavity within the molecule. The exterior region of the CD, which is populated with hydroxyl groups, is hydrophilic, whereas the cavity is dominated by hydrophobic interactions, enabling CDs to form relatively strong complexes with hydrophobic guests. The hydrophobicity of the CDs' exterior ensures their water solubility, a property that results in their being potential solubilisers [1].

The size of the molecules that can be bound by a particular CD is related to the size of the cavity. *α*-CDs bind alkyl-chains of various lengths, whilst benzene, for instance, is seen to be too large. *β*-CDs cavities can complex more bulky molecules, such as adamantane, naphthalene or various benzene derivatives. *γ*-CDs can bind annelated ring systems and even buckyballs up to C_60_. The ability of CDs to bind molecules of particular sizes has been termed 'size recognition' [1,2].

*β*-CDs in particular have proven to be in high demand in the pharmaceutical industry because their cavities seem to be almost 'predestined' to bind drug-size molecules. Several formulations are on the marketplace in which *β*-CDs are applied as solubilisers of insoluble drugs [3-7]. Other industrial applications have been reported in the food industry, where CDs have been used to protect flavours or vitamins from oxidation [8]. It is therefore felt that an ability to predict the thermodynamic properties of this particular host-guest system could potentially have a direct impact on the development of novel pharmaceutical formulations.

Some time has been spent on studying and predicting the binding free energies (Δ*G°*) of CD inclusion complexes using computational methods [9,10]. Amongst these statistical methods, those based on multiple regression [11,12] or neural nets [13] have particularly proven to lead to robust prediction models.

In this study we investigate the predictability of the thermodynamic quantities relevant to complex formation, i.e. the free energy change Δ*G°*, the change of enthalpy Δ*H° *and the change of entropy ΔS°. The study is combined with a detailed performance comparison of three different types of statistical regression methods, namely principal components regression (PCR) [14], partial least squares regression (PLSR) [14] and support vector regression with forward feature selection (SVMR/FFS) [15,16]. Whereas the first two methods are well established in the field of cheminformatics, the latter is a relatively new machine learning technique, which has been successfully applied in recent research projects [17,18]. We have also reported its application as a valuable tool for increasing hit rates in similarity based virtual screenings [19].

Our study shows that the ease of predicting Δ*G° *cannot be explained by the predictability of either Δ*H° *or Δ*S°*. We shall discuss this finding in the context of a concise analysis of the experimental accuracy of thermodynamic data.

## Results and discussion

In this study we investigated the predictability of the experimental thermodynamic data for 176 guest molecules of *β*-CD (see additional file 1: Table 1). For all molecules we had experimental values for the three fundamental thermodynamic quantities, entropy change (*TΔS°*), enthalpy change (Δ*H°*) and the Gibbs free energy of binding (Δ*G°*). Statistical models were developed to predict each of these properties on the basis of computed molecular descriptors. We applied three different types of regression methods: principal component regression (PCR), partial least squares regression (PLSR) and support vector machine regression with forward feature selection (SVMR/FFS). To validate and assess our models we performed tenfold cross-validation and nested cross-validation protocols.

**Table 1:** Comparison of the regression methods for ten-fold cross validation. The maximal *q*^2 ^values are reported for each thermodynamic parameter.

	Δ***G****°*	Δ***H****°*	***TΔS****°*
	*q*^2^(max)	*q*^2^(max)	*Q*^2^(max)
PCR	0.71	0.54	0.35
PLSR	0.74	0.53	0.31
SVMR/FFS	0.89	0.75	0.63

### Comparison of the regression methods

The results of the cross-validations are outlined in Table 1. We can express the cross-validation parameter *q*^2^, which includes the prediction errors, as:

q2=1−σ2(Δy)σ2(y),
 MathType@MTEF@5@5@+=feaafiart1ev1aaatCvAUfKttLearuWrP9MDH5MBPbIqV92AaeXatLxBI9gBaebbnrfifHhDYfgasaacPC6xNi=xI8qiVKYPFjYdHaVhbbf9v8qqaqFr0xc9vqFj0dXdbba91qpepeI8k8fiI+fsY=rqGqVepae9pg0db9vqaiVgFr0xfr=xfr=xc9adbaqaaeGacaGaaiaabeqaaeqabiWaaaGcbaGaemyCae3aaWbaaSqabeaacqaIYaGmaaGccqGH9aqpcqaIXaqmcqGHsisljuaGdaWcaaqaaGGaciab=n8aZnaaCaaabeqaaiabikdaYaaacqGGOaakcqqHuoarcqWG5bqEcqGGPaqkaeaacqWFdpWCdaahaaqabeaacqaIYaGmaaGaeiikaGIaemyEaKNaeiykaKcaaOGaeiilaWcaaa@4090@

where *σ*^2^(...) = variance of the respective quantity in brackets, Δ*y *is the deviation between prediction and experimental value, and *y *is the quantity being predicted.

On applying PCR to predict Δ*G°*, Δ*H° *and *TΔS°*, the highest cross-validation *q*^2 ^values obtained are 0.71, 0.54 and 0.35 respectively (Table 1). PLSR leads to models with maximal *q*^2 ^values for the three properties of 0.74, 0.53 and 0.31, respectively. The highest *q*^2 ^values obtained are for SVMR/FFS, namely, 0.89, 0.75 and 0.63 respectively.

**Figure 1:**
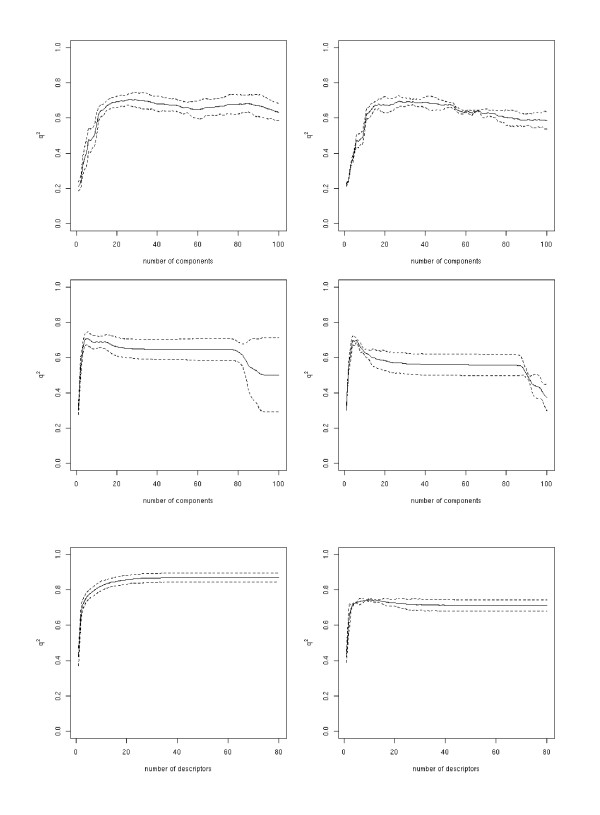
Dependence of the cross-validation coefficient *q*^2 ^(Δ*G°*) on the number of components/descriptors integrated into a model for the inner (left column) and the outer loop (right column) of the nested-cross validation for all three methods (top to bottom: PCR, PLS, and SVM).

The shape of the curve when plotting the number of components or descriptors against *q*^2 ^is characteristic for each of the regression methods (Figure 1 – the left column shows a representative example). PLSR directly steers towards the maximal *q*^2 ^value and thus reaches its peak with only a few components. After this maximum, the *q*^2 ^value decreases slightly, levels off, until dropping drastically at one point. The curves for PCR look somewhat different; the maximum *q*^2 ^is reached with significantly more components, and in-between local minima are also seen to exist. The differences in the shape of the curves can be explained by the way in which the components are obtained. While in PLSR the components are derived from the cross-covariance between the descriptors and the predictors, in PCR the components are only derived from the descriptor matrix. For SVMR/FFS the *q*^2 ^value increases continuously with each added descriptor until it plateaus at the maximal *q*^2 ^value. This continuous increase in the *q*^2 ^value is due to the FFS selection criterion, which includes the descriptor that shows the highest improvement in cross-validation performance.

In order to validate accurately the statistical models we performed the nested cross-validation protocol as described by Ruschhaupt *et al*. [20]. This type of validation gives an accurate estimate of the reliability of predicting external data. The method consists of inner and outer cross-validation loops. In the inner loop 10-fold cross-validation is used to identify the optimal settings for the complete algorithm that are then used for the outer loop. In the outer loop the performance of the best model obtained from the inner loop is tested on unseen data. Thus the test-sets in the outer loop do not in any way influence the training, with the performance of the learner on these test sets being an objective measure of its expected performance on similar data. Compared to the typical validation by external test-sets, this approach has an advantage of being less dependent on the partitioning into test and training sets, as each data point is part of the test-set exactly once. For each regression method this procedure was performed three times resulting in nine different models and prediction assessments. A more detailed description of nested cross validation is outlined in the methods section.

**Figure 2:**
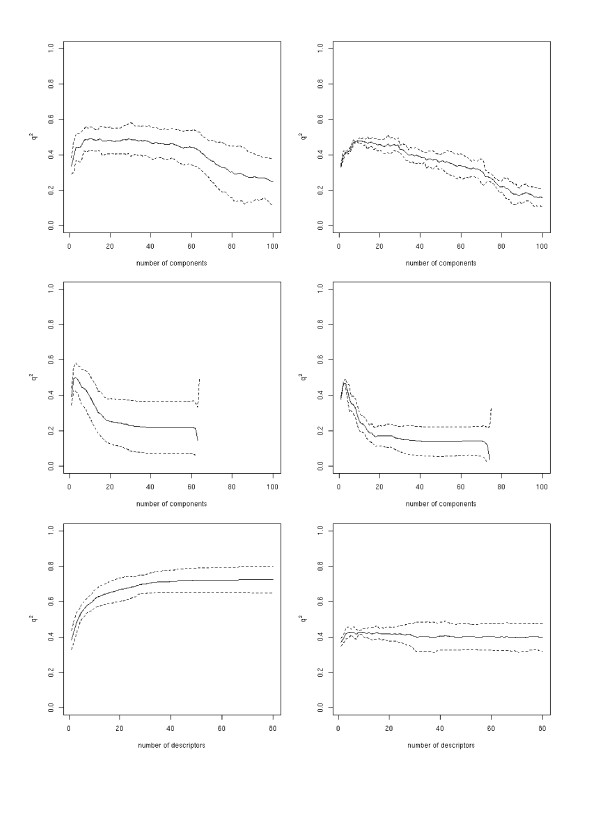
Dependence of the cross-validation coefficient *q*^2 ^(Δ*H°*) on the number of components/descriptors integrated into a model for the inner (left column) and the outer loop (right column) of the nested-cross validation for all three methods (top to bottom: PCR, PLS, and SVM).

**Figure 3:**
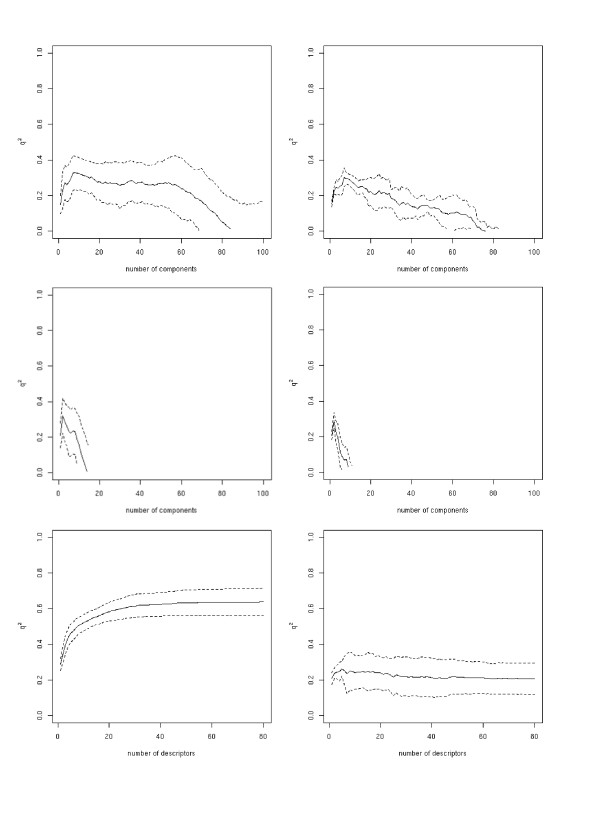
Dependence of the cross-validation coefficient *q*^2 ^(TΔ*S°*) on the number of components/descriptors integrated into a model for the inner (left column) and the outer loop (right column) of the nested-cross validation for all three methods (top to bottom: PCR, PLS, and SVM).

The PCR model predicts the molecules' Δ*G° *values in the outer loop with a *q*^2 ^of 0.69 ± 0.03 to the experimentally determined values, while PLSR gives a *q*^2 ^of 0.69 ± 0.03 and SVMR/FFS a value of 0.71 ± 0.03 (Figure 1 and Table 2). In the case of SVMR/FFS, a drastic decrease in the outer loop's *q*^2^, in comparison to that of the inner loop, can be observed. The maximal obtained *q*^2 ^value in the inner loop is 0.87, whereas in the outer loop a value of only 0.74 was found. PLSR and PCR show more stable behaviour with comparable *q*^2 ^values for the inner and the outer loops. The correlations obtained for the prediction of Δ*H° *and *TΔS° *(see Figure 2 and Table 3, and Figure 3 and Table 4 respectively) are clearly below those obtained for the prediction of Δ*G° *with all regression methods. For both Δ*H° *and *TΔS° *none of the regression methods resulted in a *q*^2 ^value of above 0.5 in the outer loop. This finding in particular highlights the risk of over-fitting the SVMR/FFS model to the data, because in the ten-fold cross-validation comparably good correlations were obtained even for Δ*H° *and *TΔS°*. The over-fitting of the SVMR/FFS model has mainly to do with the forward feature selection algorithm, which uses the squared correlation coefficient to choose the next descriptor in the iteration. Thus the execution of a nested cross validation is essential for obtaining a realistic estimate of the method's predictive ability.

**Table 2:** Comparison of the regression methods for nested cross-validation (Δ*G°*). Shown are the maximal *q*^2 ^in the inner loop (*q*^2^(max)-inner loop), the maximal *q*^2 ^in the outer loop (*q*^2^(max)-outer loop) and the *q*^2 ^of the outer loop predicted by the model with the maximal *q*^2 ^in the inner loop (*q*^2 ^(inner loop-max)-outer loop).

*Regression method*	*q*^2^*(max)-inner loop*	*q*^2^*(max)-outerloop*	*q*^2^*(inner loop-max)-outer loop*
PCR	0.71 ± 0.03	0.7 ± 0.03	0.69 ± 0.03
PLSR	0.71 ± 0.03	0.7 ± 0.01	0.69 ± 0.03
SVMR/FFS	0.87 ± 0.03	0.74 ± 0.01	0.71 ± 0.03

**Table 3:** Comparison of the regression methods for nested cross-validation (Δ*H°*) with respect to *q*^2^. Shown are the maximal *q*^2 ^in the inner loop (*q*^2^(max)-inner loop), the maximal *q*^2 ^in the outer loop (*q*^2^(max)-outer loop) and the *q*^2 ^of the outer loop predicted by the model with the maximal *q*^2 ^in the inner loop (*q*^2^(inner loop-max)-outer loop).

*Regression method*	*q*^2^*(max)-inner loop*	*q*^2^*(max)-outerloop*	*q*^2^*(inner loop-max)-outer loop*
PCR	0.49 ± 0.07	0.48 ± 0.02	0.48 ± 0.02
PLSR	0.5 ± 0.08	0.47 ± 0.02	0.47 ± 0.02
SVMR/FFS	0.73 ± 0.08	0.43 ± 0.02	0.4 ± 0.08

**Table 4:** Comparison of the regression methods for nested cross-validation (*TΔS°*) with respect to *q*^2^. Shown are the maximal *q*^2 ^in the inner loop (*q*^2^(max)-inner loop), the maximal *q*^2 ^in the outer loop (*q*^2^(max)-outer loop) and the *q*^2 ^of the outer loop predicted by the model with the maximal *q*^2 ^in the inner loop (*q*^2^(inner loop-max)-outer loop).

*Regression method*	*q*^2^*(max)-inner loop*	*q*^2^*(max)-outerloop*	*q*^2^*(inner loop-max)-outer loop*
PCR	0.33 ± 0.09	0.3 ± 0.05	0.3 ± 0.03
PLSR	0.32 ± 0.1	0.29 ± 0.04	0.29 ± 0.04
SVMR/FFS	0.64 ± 0.08	0.26 ± 0.04	0.21 ± -0.09

### Predictability of different thermodynamic quantities

**Figure 4:**
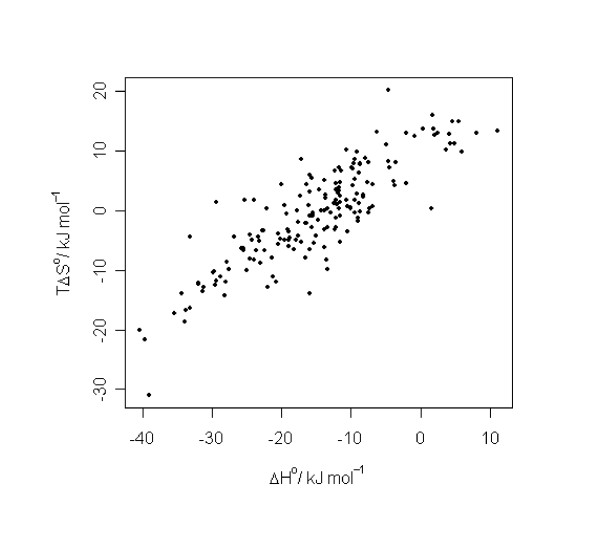
Enthalpy-entropy compensation.

The relationship between the three quantities is given, on the one hand, by classical thermodynamics (Δ*G° *= Δ*H°*-*TΔS°*), and the empirical finding of enthalpy-entropy compensation, on the other [21]. In Figure 4 we can observe the enthalpy-entropy compensation effect for the current data set. Surprisingly, for all regression methods the best predictions obtained were those for Δ*G°*. For *TΔS°*, in particular, no predictive regression models could be generated with any of the methods. One possible reason for the differing predictabilities of the three quantities has been suggested by Sharp [21]. In an analysis of the thermodynamics of three different protein systems, Sharp suggests that the most probable explanation for entropy-enthalpy compensation is the higher experimental error involved in the determination of Δ*H° *and *TΔS° *[21]. If Δ*G° *can be measured reliably, while there is significant error in the determination of Δ*H° *and *TΔS°*, the last two quantities will vary significantly and in a correlated manner, because of the relationship Δ*G° *= Δ*H°*-*TΔS°*. This explanation agrees with the apparent difficulties that we face in predicting Δ*H° *and *TΔS° *in comparison to Δ*G°*. Furthermore, it has been observed that experimental parameters have a significantly higher influence on Δ*H° *and *TΔS° *than on Δ*G°*. Ross *et al.*, for example, measured the thermodynamic properties of the complex formed from cyclohexanol and *β*-CD at four different temperatures (288 – 318 K) [22]. While the Δ*G° *values were about the same in all measurements (16.3 ± 0.2 kJmol^-1^), those for Δ*H° *varied between -2.8 and -13.0 kJmol^-1 ^and *TΔS° *13.2 and 3.6 kJmol^-1^. The stronger dependence of Δ*H° *and *TΔS° *on experimental parameters leads to larger errors, particularly when data from different laboratories are used. This was apparent in our study, and for this reason, the explanation for the existence of different experimental accuracies appears plausible.

We also analyzed differences in the thermodynamic properties of structurally related guest molecules in order to obtain a more detailed picture of the reasons behind the poor predictability of Δ*H° *and *TΔS°*. To extend the experimental data-basis of our analysis, we integrated additional data if multiple measurements for a guest molecule were listed in Rekharsky's review [23]. For those compounds for which we had independent data from other published studies, we calculated the standard deviations for Δ*G°*, Δ*H° *and *TΔS°*, and averaged these over all compounds; the respective values obtained being 1.8 kJmol^-1^, 2.1 kJmol^-1^, and 2.7 kJmol^-1^. These values describe the average absolute error in the experimental determination of the thermodynamic parameters. Interestingly, the magnitudes are entirely consistent with those obtained using the usual practice of determining entropy changes, that is, from the difference between the measured change in enthalpy and the measured change in the binding free energy. If we assume independent errors in the two latter quantities, we can calculate the expected error in the entropy change by means of the law of error propagation – the root of the sum of the squares of the errors in enthalpy and free energy is 2.8 kJmol^-1^.

It is noteworthy that the magnitudes of the experimental errors found here are higher than those generally reported. This is mainly because our data include systematic errors arising from the compilation of data from different laboratories, whose experimental protocols most likely differ. The error values certainly agree with the predictability of the three quantities. However, the error is rather low when compared to the overall spread of the corresponding quantities: the overall standard deviations of the thermodynamic parameters in our dataset are 5.3 kJmol^-1^, 9.6 kJmol^-1^, and 8.5 kJmol^-1 ^for Δ*G°*, Δ*H° *and *TΔS° *respectively. The average root mean square errors of the predicted to the experimental values obtained with SVMR/FFS are 2.8 kJmol^-1 ^(Δ*G°*), 7.5 kJmol^-1^(Δ*H°*) and 7.4 kJmol^-1 ^(*TΔS°*). While the prediction of Δ*G° *appears to be limited mainly by experimental error (prediction error 2.8 kJmol^-1 ^compared to the experimental error 1.8 kJmol^-1^), Δ*H° *and *TΔS° *are clearly poorly predicted, which cannot be explained by the slightly higher values of the experimental error alone.

**Figure 5:**
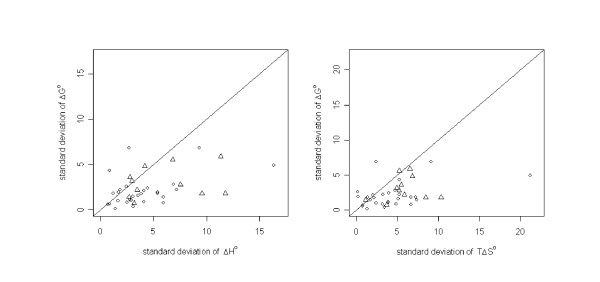
Plot of the standard deviations of Δ*G° *against the standard deviations of Δ*H° *(left side) and *TΔS° *(right side) for each cluster. Circles stand for clusters in which the experimental measurements were all performed within one laboratory. Triangles denote clusters containing data from different laboratories.

To further analyze these findings, we clustered the dataset compounds on the basis of their molecular similarity. Clusters were built using a similarity threshold of 0.7 with a complete linkage algorithm. In this way all structures within a cluster have a similarity of  (see additional file 1: Table 2). We then calculated the mean values for Δ*G°*, Δ*H° *and *TΔS° *together with the standard deviations for all molecules within a cluster. Figure 5 shows the plot of the standard deviations of Δ*G° *against the corresponding standard deviations of Δ*H° *and *TΔS° *within each cluster. In the majority of all cases, the points lie below the diagonal, indicating that the variance in the experimental Δ*H° *and *TΔS° *values is higher than the variance of the corresponding Δ*G° *values. This indicates the enthalpy and the entropy values' higher dependence on small structural changes in the ligand. This is nicely illustrated, for example, by the calorimetrically-derived thermodynamic data for inclusion complexes of a range of sulfonamides (additional file 1: Table 2 – Cluster ID 39), which were all found in one similarity cluster and studied within one laboratory. At ± 1.8 kJmol^-1^, the standard deviation of the Δ*G° *values is relatively small. The corresponding standard deviations of Δ*H° *and *TΔS°*, however, are clearly higher, at ± 5.04 kJmol^-1 ^and ± 3.84 kJmol^-1^, respectively.

In addition, we attempted a nearest-neighbour prediction of Δ*G°*, Δ*H° *and *TΔS° *using the graph-based similarity of the molecules [31]. This method is independent of the E-Dragon descriptors [29] and regression methods. For each molecule within the dataset the three thermodynamic quantities were predicted to be equal to those of the most similar compound within the set. We obtained squared correlation coefficients *r*^2 ^values of 0.50 for Δ*G°*, 0.47 for Δ*H° *and 0.29 for *TΔS°*. Except for a loss of accuracy in the prediction of Δ*G°*, the results are very similar to those obtained from the regression-based prediction. The main trend in the predictability of the thermodynamic quantities observed in the regression analysis can also be observed in this analysis, where again *TΔS° *proves to be the least predictable thermodynamic parameter.

This analysis indicates that the lower ability to predict *TΔS° *– and to a lesser extent, Δ*H° *– for different ligands has to do with the more complex dependence of *TΔS° *on even small structural changes in the ligand. This explanation is also consistent with the empirical observation of enthalpy-entropy compensation. The relative insensitivity of Δ*G° *to small structural changes compared to the other two quantities would lead to the compensation effects in enthalpy and entropy according to Δ*G° *= Δ*H°*-*TΔS°*, and conversely, given entropy-enthalpy compensation, changes in entropy would lead to smaller changes in free energy.

## Conclusion

In this study we investigated the predictability of three important thermodynamic quantities, namely, the free energy of binding, heat of formation and the entropy change upon binding. To this end, we chose *β*-cyclodextrin with its ligands, a very well-studied system for which there is a large amount of high quality binding data available. We were able to show that free energies of binding can be reliably predicted by means of simple, readily available molecular descriptors with all three of the linear regression methods studied. The SVMR/FFS method has the advantage of leading to a (partly) interpretable model with comparably few descriptors. However, in the application of SVMR/FFS it is important to perform a nested cross-validation in order to obtain a realistic impression of its generalisation ability. The predictability of Δ*G° *obviously cannot be compared directly to that of Δ*H°*, as the latter is reproduced with significantly lower accuracy by the models analyzed. We found that *TΔS° *appears almost unpredictable, with an analysis of our results in the context of further data from the literature suggesting that its poor predictability – and, to a lesser extent, that of Δ*H° *– is explained by a stronger dependence of those quantities on the complex's structural details, and to a lesser extent on the wider experimental error. This would also explain the well-documented empirical finding of entropy-enthalpy compensation. In this sense our conclusion is in disagreement with that of Sharp, which suggested that entropy-enthalpy compensation is most likely due to lower accuracy in the experimental determination of binding enthalpy and entropy.

## Methods

### Dataset choice and preparation of the molecules

We assembled a dataset consisting of 176 *β*-CD ligands (see additional file 1; Table 1). These molecules are a subset of those collected by Rekharsky et al [23]. We applied the following selection criteria:

• The availability of experimental data derived from either calorimetric (cal) or UV-spectroscopic measurements;

• The availability of Δ*G°*, Δ*H° *and *TΔS° *data;

• The exclusion of all guest molecules whose data deviated from measurements of other groups.

We drew two-dimensional Lewis structures of the molecules with ISIS-Draw and exported them as MDL MOL files [24]. The protonation state of each molecule was manually set according to the pH value at which the measurement was performed. When no pH data were available, a reasonable state was set. To generate three dimensional low-energy structures from the mol file we used CORINA [25]. We then converted the structures to SD-files [26]. Finally, all structures were manually inspected and, when needed, corrected.

### Calculation and processing of molecular descriptors

We calculated molecular descriptors for all molecules using the web service E-Dragon, which is part of the Virtual Computational Chemistry Laboratory [27,28]. E-Dragon can calculate up to 1,666 different molecular descriptors [29], which are grouped into different categories ranging from simple atom-type descriptors or fragment counts to more sophisticated topological, geometrical or quantum chemical descriptors. In order to prevent numerical problems and to ensure the avoidance of any bias in the descriptor space, we normalised all descriptor values to a range between -1 and +1.

### Regression Methods

The statistical methods used in this work have been employed on numerous occasions elsewhere. For PCR and Partial PLSR the R-package PLS was used [14]. The support vector machine regression was performed with LIBSVM, which was developed by Chang *et al *[30].

### Principal component regression

In PCR a multiple linear regression is performed on principal components. Principal components are linear combinations of the descriptors in the data matrix and explain their variance. They are derived from the covariance matrix of the calculated descriptors. The number of principal components corresponds to the data matrix rank. Its maximal value is the minimum of the number of data points (i.e. molecules) and descriptors.

The first principal component of a data matrix points in the direction that maximizes the variance of the descriptors and corresponds to the largest eigenvalue of the covariance matrix. The second corresponds to the second largest eigenvalue and points in the direction that maximizes the variance and is orthogonal to the first principal component, and so on for the remaining principal components. The PCR model is generated on a subset of the components. The subset is built by selecting the components in the order of their ability to explain the variance in the dependent variable, i.e. in this study, the thermodynamic properties.

### Partial Least Squares Regression

PLSR is very similar to PCR, however, while the covariance matrix of the data is used to generate the principal components, in PLSR the principal components are derived from the cross-covariance between the data matrix and the dependent variables. Hence, while in PCR the eigenvectors of the data covariance matrix are used to span the solution space, in PLSR the directions of maximal covariance between data and the dependent variables are used.

### Support vector machine regression combined with forward feature selection

The theoretical background of support vector machine regression has been described in detail by Drucker *et al *[15]. Support vector regression is a straightforward variant of support vector machine (SVM) classification [16]. In classification problems SVMs find the hyper plane that separates positive examples from negative examples with a maximum margin (where the margin is defined as the distance of the closest data point from the separating hyper plane). In this way a statistical model is produced that only depends on a subset of the training data, namely those data points that are close enough to influence the size of the margin and the orientation of the hyper plane. These are the most difficult examples in the training set. They are termed 'support vectors' because they define the orientation of the separating plane. In support vector regression (SVMR) the same effect (namely that the final model depends only on a subset of the data) is achieved by the use of a so-called '*ε*-insensitive cost function', which during model optimization ignores errors up to a defined threshold. This means that any training data being predicted by the current model with an accuracy of up to *ε *can be neglected.

In this work we added a 'forward feature selection procedure', which is in some respects similar to the component extension in PCR and PLSR. Forward feature selection increases the learning performance and the interpretability of the regression model as only descriptors are selected that significantly improve the SVMR model. The selection of descriptors produces combinatorial explosion if all possible subsets of all the available descriptors have to be considered. This, of course, is not feasible if the number of descriptors is too large. To overcome this problem, forward feature selection uses the following greedy heuristic, that is, for each single descriptor a support vector regression model is trained with tenfold cross-validation. The descriptor leading to the model with the highest *q*^2 ^value is selected as the start descriptor. The procedure is repeated to find the next descriptor to form the best pair, triplet etc., iteratively expanding the model by one descriptor until *q*^2 ^reaches a maximum, at which point the final model is obtained.

### Validation by means of nested cross-validation

In order to validate whether our model generation procedures can lead to a predictive model that provides reliable output, we performed a nested three-way cross-validation protocol as proposed by Ruschhaupt *et al *[20] for each of the regression methods. To this end, we first split the training set into three equally sized subsets by randomly assigning training dataset molecules to one of the subsets (S_1 _and S_2 _consist of 59 molecules, S_3 _consists of 58 molecules). We then generated three validation sets, each as a combination of two subsets (V_1_→ S_1 _and S_2_, V_2_→ S_1 _and S_3_, V_3_→ S_2 _and S_3_), such that each of the validation sets could be used as a training-set for predicting the binding energies of the remaining subset (test set) that is not included in the respective training set. A ten-fold cross validation is used within the validation set (e.g. V_1_) to identify the optimal model (e.g. the number of components in PCR/PLS or descriptors in SVMR), meaning that V_1 _is again separated into ten subsets that are used for cross validation in a ten-fold loop. The model resulting from this inner loop is then used to predict the test set (e.g. S_3_). This is performed once for every validation set/test set combination, three times in total. Thus the reported results on the test sets from the outer loop do not in any way influence the choice of model parameters and are comparable to independent test set validations. The advantage of this approach over independent test set validation, is that every data point is predicted once in one of the three test sets, thus reducing the effects of test set choice.

### Calculation of molecular similarity and clustering of the molecules

To cluster the dataset molecules and for the nearest-neighbour prediction, we calculated all pair-wise molecular similarities using our in-house similarity tool GMA [31]. The molecular similarity was calculated on the basis of a graph-based alignment. The better the molecular graphs (i.e. the topology and the atom types) of two molecules can be matched, the greater the similarity between these two molecules (1 = identical, 0 = dissimilar). On the basis of these similarities we performed complete-linkage hierarchical clustering. The cluster tree was cut off at a similarity threshold of 0.7. Hence, within one cluster only those molecules that exhibit a similarity of 0.7 or higher are grouped (see additional file 1, Table 2).

## Authors' contributions

The study was designed performed and analyzed by JA and AS. Both authors approved the final manuscript.

## Supplementary Material

Additional file 1 Contains two tables with all the data used in this study (Table 1) and a list of the clusters' average properties according to structural similarity (Table 2). Click here for file
