# Supplementary material for: On the ease of predicting the thermodynamic properties of beta-cyclodextrin inclusion complexes
Source: Chem Cent J. 2007 Nov 15;1:29. doi: 10.1186/1752-153X-1-29 (PMC2228290; doi:10.1186/1752-153X-1-29)
Supplement: Additional file 1 — Contains two tables with all the data used in this study (Table 1) and a list of the clusters' average properties according to structural similarity (Table 2). [file 1752-153X-1-29-S1.pdf]

Table 1 shows the assembled dataset for the generation of the QSPR models. For each molecule the three thermodynamic parameters ΔG°, ΔH°, and $T\Delta S^\circ$ for the complex formation with β-cyclodextrin are listed. The data for each of the molecules was taken from (Rekharsky and Inoue, 1998).

Table 1: The data used for the generation of the QSPR models. All experimental values were taken from Rekharsky and Inoue (1998).

**Molecule**	ΔG° [kJ mol^{-1}]	ΔH° [kJ mol^{-1}]	$T\Delta S^\circ$ [kJ mol^{-1}]
(+-)-cis-2-methylcyclohexanol	-17.08	-9.9	7.2
(+-)-norphenylephrine	-8.65	-20.7	-12
(+-)-octopamine	-9.4	-15.86	-6.5
(+-)-trans-2-methylcyclohexanol	-16.38	-8.66	7.72
(-)-anisodamine	-13.3	-17.6	-4.3
(-)-anisodine	-10.6	-11.6	-1
(-)-atropine	-14.6	-19.5	-4.9
(-)-scopolamine	-12.9	-17.9	-5
(1-methylhexyl)ammonium	-10.7	2	12.7
(1R,2R)-(-)-pseudoephedrine	-10.49	-9.99	0.5
(1S,2R)-(+)-ephedrine	-9.95	-8.79	1.2
(2,5-dimethoxyphenethyl)-ammonium	-9.08	-9.39	-0.3
(2-methoxyphenethyl)ammonium	-5.15	-13.5	-8.3
(3,4-dihydroxyphenethyl)-ammonium	-8.58	-16.52	-7.9
(3,4-dimethoxyphenethyl)-ammonium	-6.52	-2	4.5
(3-methoxyphenethyl)ammonium	-10.39	-13.32	-2.9
(3-methylphenyl)acetate	-6.1	-11.5	-5.4
(3-phenylpropyl)ammonium	-11.29	-9.44	1.8
(4-hydroxyphenethyl)ammonium	-10.58	-13.8	-3.2
(4-methoxyphenethyl)ammonium	-10.78	-8.21	2.6
(4-methylphenethyl)ammonium	-11.11	-6.84	4.3
(4-methylphenyl)acetate	-9.17	-12.1	-2.9
(R)-(-)-2-butanol	-6.4	4.9	11.3
(R)-(-)-2-hexanol	-11.8	1.9	13.7
(R)-(-)-phenylephrine	-9.1	-21.9	-12.8
(S)-(+)-2-pentanol	-8.6	4.1	12.8
1-adamantaneacetate	-28.7	-33.1	-4.4
1-adamantaneammonium	-22.4	-22.1	0.3
1-adamantanecarboxylate	-25.7	-23.9	1.8
1-adamantylmethylammonium	-25.5	-17.2	8.6
1-adamantyltrimethylammonium	-20.5	-24.5	-4
1-benzylimidazole	-14.92	-15.9	-1
1-bicyclo[2.2.1]hept-2-enecarboxylate	-15.7	-7.5	8.1
1-bicyclo[2.2.1]heptanecarboxylate	-16.7	-8	8.8
1-bicyclo[2.2.2]octanecarboxylate	-21.9	-15.9	5.9
1-butanol	-6.9	4.3	11.2
1-butylimidazole	-12.5	-10.7	1.8
1-hexanol	-13.3	0.4	13.7
1-methylcyclohexanol	-17.47	-9.6	7.9
1-naphthaleneacetate	-24.8	-4.6	20.2
1-naphthalenesulfonate	-19.4	-6.2	13.2
1-pentanol	-10.3	4.6	14.9

. . . continued on next page

Table 1 . . . continued from previous page

**Molecule**	ΔG°	ΔH°	$T\Delta S^\circ$
1-phenylimidazole	-8	-39	-31
1-propanol	-3.7	6	9.8
2,2-dimethyl-1-propanol	-15.5	-8.8	6.3
2,3,6-naphthalenetrisulfonate	-12.7	-12.9	-0.3
2,6-naphthalenedisulfonate	-18.8	-11.7	7.1
2,7-naphthalenedisulfonate	-13.9	-28.2	-14.3
2-(4-aminophenyl)-ethyl-ammonium	-8.54	-8.7	-0.2
2-chlorophenol	-13.1	-19	-6
2-methylcyclohexanone	-15.7	-13.7	2.1
2-norbornaneacetate	-20.8	-10.7	10.2
2-propanol	-2.4	11.1	13.4
3-(2-hydroxyphenyl)propionate	-10.89	-15.1	-4.2
3-(4-hydroxyphenyl)propionate	-14.11	-14.2	-0.1
3-O-methyldopamine	-3.6	-13.4	-9.8
3-chlorophenol	-13.1	-19	-5
3-methoxyphenylacetate	-9.02	-12.3	-3.2
3-methylcyclohexanol	-16.66	-8.74	7.93
3-nitrophenol	-13.9	-12.1	1.8
3-noradamantanecarboxylate	-21.1	-15.7	5.4
3-phenylbutanoate	-14.72	-9.41	5.3
4-O-methyldopamine	-9.78	-15.3	-5.5
4-amino-1-naphthalenesulfonate	-9.7	-10	0.3
4-benzylpiperidine	-18.83	-13.8	5.1
4-bromophenol	-16.7	-12.2	4.5
4-chlorophenol	-14.9	-11.9	3
4-hydroxycoumarin	-13.1	-12	1.1
4-iodophenol	-17	-16.1	0.9
4-methoxyphenylacetate	-10.51	-8.22	2.3
4-methylphenol	-13.7	-12.5	1.2
4-nitrophenol	-13.8	-13.4	0.3
4-phenylbutanoate	-15.06	-11.78	3.3
5-methylresorcinol	-9.8	-21.2	-11.2
6-[(4-tert-butylphenyl)-amino]-2-naphthalenesulfonate	-27.1	-25.3	1.8
D-glucose	1.3	1.6	0.3
L-alpha-O-benzylglycerol	-12.03	-9.2	2.8
L-phenylalanine	-7.2	-9	-1.8
L-phenylalanineamide	-7.7	-9	-1.3
L-tryptophan	-13.3	-0.8	12.5
L-tyrosine	-8.7	-3.8	4.9
N-methylphenethylammonium	-7.59	-7.3	0.3
acenocoumarin	-14.7	-15.5	-0.7
adiphenine	-19.6	-31.9	-12.3
amobarbital	-17.7	-17.8	-0.1
aspartame	-12	-11.7	0.3
barbital	-13.9	-11.5	2.4
benzene	-11.6	-3.5	8.1
benzoate	-6.86	-10.5	-3.6
benzylalcohol	-7.7	-13.8	-6.2
bromodiphenhydramine	-19	-25.4	-6.4

. . . continued on next page

Table 1 . . . continued from previous page

**Molecule**	ΔG°	ΔH°	$T\Delta S^{\circ}$
butabarbital	-16.9	-33.2	-16.4
butethal	-16.8	-9.8	7
butylbarbituricacid	-14.8	-15.8	-1.0
butylthiobarbituricacid	-16.4	-20.4	-3.9
chlorcyclizine	-19.4	-22.8	-3.4
chlorpromazine	-22.4	-26.8	-4.5
cinnarizine	-19.8	-17.3	2.4
cis-1,2-cyclohexanediol	-13.9	-9.8	4.2
cis-4-methylcyclohexanol	-18.07	-9.5	8.55
cyclizine	-17.6	-28.8	-11.2
cyclobarbital	-17.9	-20.2	-4.8
cyclobutanol	-6.5	3.7	10.2
cycloheptanol	-19.08	-12.37	6.7
cyclohexanol	-16.2	-4.9	11.1
cyclohexanone	-15.5	-11.7	3.9
cyclooctanol	-20.8	-16.4	4.4
cyclopentanol	-12.76	-4.56	8.2
di-2-(1-adamantyl)ethylhydrogen-phosphate	-30.5	-29.3	1.3
dicumarol	-20.5	-40.4	-20
diphenhydramine	-17.5	-29.4	-11.9
diphenidol	-17	-33.7	-16.7
diphenylpyraline	-19.1	-27.8	-8.7
ethylthiobarbituricacid	-14.0	-15.5	-0.3
flufenamicacid	-18.1	-11.4	6.7
flurbiprofen	-18.8	-23.3	-4.5
heptanoate	-14.2	1.8	15.9
heptylbarbituricacid	-19.8	-32.0	-12.2
hexanoate	-9.54	5.5	15
hexobarbital	-16.4	-24.5	-8.2
hexylammonium	-10.4	2.5	12.9
hexylthiobarbituricacid	-20.0	-29.6	-10.2
hydroquinone	-11.7	-17.1	-5.4
hydroxyzine	-19.2	-24.2	-5
imidazole	-1.6	-16	-14
meclizine	-19.1	-22.6	-3.4
mephobarbital	-18.1	-39.7	-21.6
methapyriline	-14.5	-15.5	-1
methylorange (anion)	-18.8	-15.9	3.1
methylred (anion)	-20.5	-19.6	0.9
niflumicacid	-15.5	-19	-3.5
octylammonium	-15	-2	13
orphenadrine	-17.7	-31.3	-13.5
pentanoate	-5.3	8	13
pentobarbital	-18	-23.2	-5.2
pentylthiobarbituricacid	-19.2	-23.6	-6.7
phenethylammonium	-7.43	-6.9	0.6
phenobarbital	-18.3	-31.2	-12.9
phenol	-11.3	-12.2	-1.2
phenprocoumon	-16.2	-13.6	2.6

. . . continued on next page

Table 1 ... continued from previous page

**Molecule**	ΔG°	ΔH°	$T\Delta S^{\circ}$
phenylacetate	-7.1	-7.5	-0.4
phenylpropionate	-12.27	-7.6	4.7
piroxicam	-11.2	-10.5	0.7
proadifen	-16.9	-29.5	-12.6
propylbarbituricacid	-12.8	-11.6	1.3
propylthiobarbituricacid	-14.1	-16.4	-2.2
prostaglandin E2	-18.7	-19.3	-0.6
resorcinol	-11.6	-18.2	-6.5
secobarbital	-18.6	-25.4	-6.7
sulfadiazine	-15.7	-24	-8.3
sulfadimethoxine	-15.92	-19.1	-3.2
sulfaethidole	-18	-14.6	3.5
sulfaisoxazole	-15.95	-28	-12
sulfamerazine	-14.44	-16.5	-2.1
sulfamethizole	-17.7	-27.6	-9.9
sulfamethoxazole	-15.81	-22.5	-6.7
sulfapyridine	-15.2	-33.9	-18.7
sulfathiazole	-19.24	-29.8	-10.5
sulfathidole	-18.1	-35.4	-17.3
sulfoisomidine	-12.84	-15.6	-2.8
terfenadine	-24.4	-20	4.4
thenyldiamine	-13.2	-14.8	-1.6
thiopental	-19.6	-25.7	-6.3
thiophenobarbital	-20.5	-34.4	-14.0
trans-1,2-cyclohexanediol	-11.4	-4.4	7.2
trans-2-hydroxycinnamic acid	-14.7	-23.0	-8.9
trans-2-methylcinnamic acid	-14.9	-12.1	3.5
trans-3-hydroxycinnamate	-13.5	-21.3	-8.0
trans-3-methylcinnamate	-14.4	-18.8	-4.6
trans-4-hydroxycinnamate	-14.9	-20.5	-5.6
trans-4-methylcinnamate	-15.2	-17.6	-1.9
trans-4-methylcyclohexanol	-19	-9.1	9.9
triprolidine	-13.8	-13.8	0
tropicamide	-15	-25	-10
valerolactam	-8	-3.7	4.2
warfarin	-16.3	-11.6	4.8

In order to investigate the influence of structural changes to thermodynamical parameters we clustered the β-cyclodextrin guest molecules presented in Rekharsky and Inoue (1998) according to molecular similarity as calculated by FUZZEE. Clusters were built using a similarity threshold of 0.7 with a complete linkage algorithm. In this way all structures within a cluster have a similarity of 0.7 or higher and thus are structurally closely related compounds. In Table 2 the clusters as well as the mean values for ΔG°, ΔH° and $T\Delta S^{\circ}$ together with their standard deviations for all molecules of the cluster are given. Only clusters containing multiple data points were considered.

Table 2: The generated structural clusters are shown as used in the discussion of our paper. Only clusters containing multiple data points are shown. The experimental data is taken from reference Rekharsky and Inoue (1998).

**ID**	**Molecules**	
**Cluster-ID**	1	
**Molecules**	(+-)-cis-2-methylcyclohexanol	(+-)-trans-2-methylcyclohexanol
	(R)-(-)-2-butanol	(R)-(-)-2-butanol
	(R)-(-)-2-hexanol	(S)-(+)-2-pentanol
	1-hexanol	1-methylcyclohexanol
	1-pentanol	1-propanol
	2,2-dimethyl-1-propanol	2-propanol
	3-methylcyclohexanol	cis-4-methylcyclohexanol
	cyclobutanol	cycloheptanol
	cyclohexanol	cyclooctanol
	cyclopentanol	trans-4-methylcyclohexanol
$ø(\Delta G^{\circ}) = -14.81 \pm 5.55$	$ø(\Delta H^{\circ}) = -3.26 \pm 6.81$	$ø(T\Delta S^{\circ}) = 11.55 \pm 5.23$
**Cluster-ID**	2	
**Molecules**	(+-)-norphenylephrine	(1R,2R)-(-)-pseudoephedrine
	(1S,2R)-(+)-ephedrine	(R)-(-)-phenylephrine
$ø(\Delta G^{\circ}) = -9.62 \pm 0.74$	$ø(\Delta H^{\circ}) = -15.39 \pm 5.95$	$ø(T\Delta S^{\circ}) = -5.74 \pm 6.62$
**Cluster-ID**	3	
**Molecules**	(+-)-octopamine	(3,4-dihydroxyphenethyl)-ammonium
	3-O-methyldopamine	
$ø(\Delta G^{\circ}) = -7.63 \pm 2.4$	$ø(\Delta H^{\circ}) = -13.91 \pm 4.42$	$ø(T\Delta S^{\circ}) = -6.25 \pm 3.92$
**Cluster-ID**	4	
**Molecules**	(-)-anisodamine	(-)-anisodine
	(-)-atropine	(-)-scopolamine
$ø(\Delta G^{\circ}) = -12.92 \pm 1.45$	$ø(\Delta H^{\circ}) = -16.92 \pm 3.06$	$ø(T\Delta S^{\circ}) = -4.0 \pm 1.7$
**Cluster-ID**	5	
**Molecules**	(1-methylhexyl)ammonium	hexylammonium
	octylammonium	
$ø(\Delta G^{\circ}) = -12.03 \pm 2.57$	$ø(\Delta H^{\circ}) = 0.83 \pm 2.47$	$ø(T\Delta S^{\circ}) = 12.87 \pm 0.15$
**Cluster-ID**	6	
**Molecules**	(2,5-dimethoxyphenethyl)-ammonium	
	2-methoxyphenethyl)ammonium	
	(3-methoxyphenethyl)ammonium	
$ø(\Delta G^{\circ}) = -8.86 \pm 2.22$	$ø(\Delta H^{\circ}) = -11.62 \pm 1.83$	$ø(T\Delta S^{\circ}) = -2.76 \pm 3.24$
**Cluster-ID**	7	
**Molecules**	(3,4-dimethoxyphenethyl)-ammonium	

. . . continued on next page

Table 2 . . . continued from previous page

	4-O-methyldopamine	
ø(ΔG°) = -9.0 ± 2.2 ø(ΔH°) = -10.24 ± 7.2 ø($T\Delta S^\circ$) = -1.4 ± 5.24		
**Cluster-ID**	8	
**Molecules**	(3-methylphenyl)acetate	(4-methylphenyl)acetate
	1-naphthaleneacetate	3-phenylbutanoate
	4-phenylbutanoate	phenylacetate
	phenylpropionate	trans-2-methylcinnamicacid
	trans-3-methylcinnamate	trans-4-methylcinnamate
ø(ΔG°) = -13.41 ± 4.82 ø(ΔH°) = -10.79 ± 4.17 ø($T\Delta S^\circ$) = 2.71 ± 6.83		
**Cluster-ID**	9	
**Molecules**	(3-phenylpropyl)ammonium	(4-methylphenethyl)ammonium
	N-methylphenethylammonium	phenethylammonium
ø(ΔG°) = -9.49 ± 1.84 ø(ΔH°) = -7.45 ± 1.21 ø($T\Delta S^\circ$) = 2.05 ± 1.35		
**Cluster-ID**	10	
**Molecules**	(4-hydroxyphenethyl)ammonium	(4-methoxyphenethyl)ammonium
ø(ΔG°) = -10.68 ± 0.36 ø(ΔH°) = -11.54 ± 3.1 ø($T\Delta S^\circ$) = -0.86 ± 3.44		
**Cluster-ID**	11	
**Molecules**	1-adamantaneacetate	1-adamantanecarboxylate
	1-bicyclo[2.2.2]octanecarboxylate	2-norbornaneacetate
	3-noradamantanecarboxylate	heptanoate
	hexanoate	pentanoate
	1-bicyclo[2.2.1]hept-2-enecarboxylate	
	1-bicyclo[2.2.1]heptanecarboxylate	
ø(ΔG°) = -20.99 ± 5.91 ø(ΔH°) = -16.87 ± 11.36 ø($T\Delta S^\circ$) = 4.1 ± 6.52		
**Cluster-ID**	12	
**Molecules**	1-adamantaneammonium	1-adamantylmethylammonium
ø(ΔG°) = -23.95 ± 2.19 ø(ΔH°) = -19.65 ± 3.46 ø($T\Delta S^\circ$) = 4.45 ± 5.87		
**Cluster-ID**	13	
**Molecules**	1-adamantyltrimethylammonium	
ø(ΔG°) = -19.0 ± 2.12 ø(ΔH°) = -21.6 ± 4.1 ø($T\Delta S^\circ$) = -2.6 ± 1.98		
**Cluster-ID**	14	
**Molecules**	1-benzylimidazole	1-phenylimidazole
ø(ΔG°) = -11.46 ± 4.89 ø(ΔH°) = -27.45 ± 16.33 ø($T\Delta S^\circ$) = -16.0 ± 21.21		
**Cluster-ID**	15	
**Molecules**	1-naphthalenesulfonate	4-amino-1-naphthalenesulfonate
ø(ΔG°) = -14.55 ± 6.86 ø(ΔH°) = -8.1 ± 2.69 ø($T\Delta S^\circ$) = 6.75 ± 9.12		
**Cluster-ID**	16	
**Molecules**	2,3,6-naphthalenetrisulfonate	2,6-naphthalenedisulfonate
ø(ΔG°) = -15.75 ± 4.31 ø(ΔH°) = -12.3 ± 0.85 ø($T\Delta S^\circ$) = 3.4 ± 5.23		
**Cluster-ID**	17	
**Molecules**	2-chlorophenol	3-chlorophenol
	4-bromophenol	4-chlorophenol
	4-iodophenol	4-methylphenol
	benzylalcohol	phenol
ø(ΔG°) = -14.02 ± 3.18 ø(ΔH°) = -14.37 ± 2.97 ø($T\Delta S^\circ$) = -0.18 ± 4.91		
**Cluster-ID**	18	
**Molecules**	2-methylcyclohexanone	cyclohexanone

. . . continued on next page

Table 2 … continued from previous page

$\o(\Delta G^\circ) = -15.6 \pm 0.14$	$\o(\Delta H^\circ) = -12.7 \pm 1.41$	$\o(T\Delta S^\circ) = 3.0 \pm 1.27$
**Cluster-ID**	19	
**Molecules**	3-(2-hydroxyphenyl)propionate	4-nitrophenol
	trans-2-hydroxycinnamicacid	
$\o(\Delta G^\circ) = -14.17 \pm 1.79$	$\o(\Delta H^\circ) = -18.9 \pm 11.78$	$\o(T\Delta S^\circ) = -4.7 \pm 10.32$
**Cluster-ID**	20	
**Molecules**	3-(4-hydroxyphenyl)propionate	L-tyrosine (protonated)
	trans-4-hydroxycinnamate	
$\o(\Delta G^\circ) = -12.44 \pm 2.77$	$\o(\Delta H^\circ) = -11.3 \pm 7.54$	$\o(T\Delta S^\circ) = 1.23 \pm 5.22$
**Cluster-ID**	21	
**Molecules**	3-methoxyphenylacetate	4-methoxyphenylacetate
$\o(\Delta G^\circ) = -9.77 \pm 1.05$	$\o(\Delta H^\circ) = -10.26 \pm 2.88$	$\o(T\Delta S^\circ) = -0.45 \pm 3.89$
**Cluster-ID**	22	
**Molecules**	3-nitrophenol	hydroquinone
$\o(\Delta G^\circ) = -12.8 \pm 1.56$	$\o(\Delta H^\circ) = -14.6 \pm 3.54$	$\o(T\Delta S^\circ) = -1.8 \pm 5.09$
**Cluster-ID**	23	
**Molecules**	5-methylresorcinol	resorcinol
$\o(\Delta G^\circ) = -10.93 \pm 0.99$	$\o(\Delta H^\circ) = -20.07 \pm 1.63$	$\o(T\Delta S^\circ) = -9.07 \pm 2.38$
**Cluster-ID**	24	
**Molecules**	L-phenylalanine	L-phenylalanineamide
$\o(\Delta G^\circ) = -8.22 \pm 3.58$	$\o(\Delta H^\circ) = -9.42 \pm 2.78$	$\o(T\Delta S^\circ) = -1.2 \pm 5.47$
**Cluster-ID**	25	
**Molecules**	acenocoumarin	warfarin
$\o(\Delta G^\circ) = -15.5 \pm 1.13$	$\o(\Delta H^\circ) = -13.55 \pm 2.76$	$\o(T\Delta S^\circ) = 2.05 \pm 3.89$
**Cluster-ID**	26	
**Molecules**	adiphenine	proadifen
$\o(\Delta G^\circ) = -18.25 \pm 1.91$	$\o(\Delta H^\circ) = -30.7 \pm 1.7$	$\o(T\Delta S^\circ) = -12.45 \pm 0.21$
**Cluster-ID**	27	
**Molecules**	amobarbital	barbital
	butabarbital	butethal
	butylbarbituricacid	cyclobarbital
	heptylbarbituricacid	hexobarbital
	mephobarbital	pentobarbital
	phenobarbital	propylbarbituricacid
	secobarbital	
$\o(\Delta G^\circ) = -17.11 \pm 1.78$	$\o(\Delta H^\circ) = -24.07 \pm 9.57$	$\o(T\Delta S^\circ) = -7.05 \pm 8.46$
**Cluster-ID**	28	
**Molecules**	benzoate	
$\o(\Delta G^\circ) = -6.33 \pm 0.75$	$\o(\Delta H^\circ) = -12.75 \pm 3.18$	$\o(T\Delta S^\circ) = -6.2 \pm 3.68$
**Cluster-ID**	29	
**Molecules**	bromodiphenhydramine	diphenhydramine
	diphenylpyraline	orphenadrine
$\o(\Delta G^\circ) = -18.33 \pm 0.84$	$\o(\Delta H^\circ) = -28.48 \pm 2.5$	$\o(T\Delta S^\circ) = -10.13 \pm 3.19$
**Cluster-ID**	30	
**Molecules**	butylthiobarbituricacid	ethylthiobarbituricacid
	hexylthiobarbituricacid	pentylthiobarbituricacid

… continued on next page

	propylthiobarbituricacid	thiopental
	thiophenobarbital	
$\varnothing(\Delta G^\circ) = -17.69 \pm 2.81$	$\varnothing(\Delta H^\circ) = -23.66 \pm 6.89$	$\varnothing(T\Delta S^\circ) = -6.23 \pm 4.71$
**Cluster-ID**	31	
**Molecules**	chlorcyclizine	cinnarizine
	cyclizine	meclizine
$\varnothing(\Delta G^\circ) = -19.06 \pm 0.85$	$\varnothing(\Delta H^\circ) = -23.12 \pm 4.11$	$\varnothing(T\Delta S^\circ) = -4.06 \pm 4.85$
**Cluster-ID**	32	
**Molecules**	cis-1,2-cyclohexanediol	trans-1,2-cyclohexanediol
$\varnothing(\Delta G^\circ) = -12.65 \pm 1.77$	$\varnothing(\Delta H^\circ) = -7.1 \pm 3.82$	$\varnothing(T\Delta S^\circ) = 5.7 \pm 2.12$
**Cluster-ID**	33	
**Molecules**	di-2-(1-adamantyl)ethylhydrogenphosphate	
$\varnothing(\Delta G^\circ) = -25.65 \pm 6.86$	$\varnothing(\Delta H^\circ) = -22.7 \pm 9.33$	$\varnothing(T\Delta S^\circ) = 3.0 \pm 2.4$
**Cluster-ID**	34	
**Molecules**	flufenamicacid	niflumicacid
$\varnothing(\Delta G^\circ) = -16.8 \pm 1.84$	$\varnothing(\Delta H^\circ) = -15.2 \pm 5.37$	$\varnothing(T\Delta S^\circ) = 1.6 \pm 7.21$
**Cluster-ID**	35	
**Molecules**	flurbiprofen	
$\varnothing(\Delta G^\circ) = -19.8 \pm 1.41$	$\varnothing(\Delta H^\circ) = -19.1 \pm 5.94$	$\varnothing(T\Delta S^\circ) = 0.7 \pm 7.35$
**Cluster-ID**	36	
**Molecules**	imidazole	
$\varnothing(\Delta G^\circ) = -2.0 \pm 0.57$	$\varnothing(\Delta H^\circ) = -15.5 \pm 0.71$	$\varnothing(T\Delta S^\circ) = -13.5 \pm 0.71$
**Cluster-ID**	37	
**Molecules**	methapyriline	thenyldiamine
	triprolidine	
$\varnothing(\Delta G^\circ) = -13.83 \pm 0.65$	$\varnothing(\Delta H^\circ) = -14.7 \pm 0.85$	$\varnothing(T\Delta S^\circ) = -0.87 \pm 0.81$
**Cluster-ID**	38	
**Molecules**	methylorange (anion)	
$\varnothing(\Delta G^\circ) = -19.8 \pm 1.41$	$\varnothing(\Delta H^\circ) = -17.8 \pm 2.69$	$\varnothing(T\Delta S^\circ) = 2.3 \pm 1.13$
**Cluster-ID**	39	
**Molecules**	sulfadiazine	sulfaisoxazole
	sulfamerazine	sulfamethizole
	sulfamethoxazole	sulfapyridine
	sulfathiazole	sulfoisomidine
$\varnothing(\Delta G^\circ) = -15.98 \pm 1.92$	$\varnothing(\Delta H^\circ) = -22.91 \pm 5.04$	$\varnothing(T\Delta S^\circ) = -6.94 \pm 3.84$

References

Rekharsky, M. V. and Inoue, Y. (1998). Complexation thermodynamics of cyclodextrins. *Chem. Rev.*, 98:1875.
